
JOURNAL INFORMATION
==============================
NLM Title Abbreviation: Wirtschaftsdienst
Journal ID: Wirtschaftsdienst
NLM Journal ID: 101086612

Wirtschaftsdienst (Hamburg, Germany : 1949)

ISSN: 0043-6275

ARTICLE INFORMATION
==============================
PMCID: PMC7560784
PMID: 33082603
DOI: 10.1007/s10273-020-2734-z
Article ID: 2734
Article version: 1
Subjects: Zeitgespräch

Räumliche Flexibilisierung durch zunehmende Homeoffice-Nutzung

Garnadt Niklas niklas.garnadt@svr-wirtschaft.de
1
Schnitzer Monika Schnitzer@econ.lmu.de
2
Viete Steffen steffen.viete@svr-wirtschaft.de
1
1 grid.432326.2 0000 0001 1482 0825 Statistisches Bundesamt, Gustav-Stresemann-Ring 11, 65189 Wiesbaden, Deutschland
2 grid.5252.0 0000 0004 1936 973X Seminar f. Komparative Wirtschaftsforschung, Ludwig-Maximilians-Universität München, Akademiestr. 1, 80799 München, Deutschland
Electronic publication date: 2020 Sep 25
Print publication date: 2020
Volume: 100
Issue: 9
First page: 661
Last page: 666
Copyright: © Der/die Autor(en) 2020
License: Dieser Artikel wird unter der Creative Commons Namensnennung 4.0 International Lizenz (https://creativecommons.org/licenses/by/4.0/deed.de) veröffentlicht.
Open Access wird durch die ZBW — Leibniz-Informationszentrum Wirtschaft gefördert.
License URL: https://creativecommons.org/licenses/by/4.0/

issue-copyright-statement: © The Author(s) 2020

==============================
Prof. Dr. Monika Schnitzer ist Inhaberin des Lehrstuhls für Komparative Wirtschaftsforschung an der Ludwig-Maximilians-Universität München und Mitglied des Sachverständigenrates zur Begutachtung der gesamtwirtschaftlichen Entwicklung.

1 Der Begriff Homeoffice ist umgangssprachlich geprägt (Benkert, 2019) und kann sowohl als Mobilarbeit im weiteren Sinne als auch als Telearbeit nach § 2 Abs. 7 ArbStättV verstanden werden. Telearbeitsplätze sind vom Arbeitgeber fest eingerichtete Bildschirmarbeitsplätze im Privatbereich der Beschäftigten, wogegen Mobilarbeit sowohl im Privatbereich der Beschäftigten als auch an anderen Orten, etwa in Coworking Spaces stattfinden kann. In diesem Artikel wird Homeoffice als Mobilarbeit im weiteren Sinne verstanden.

Konzepte für räumlich flexible, durch die Digitalisierung gestützte Arbeitsmodelle bestehen bereits seit den 1970er Jahren (Nilles, 1975). Trotz deutlicher Verbesserungen der technischen Voraussetzungen verläuft deren Nutzung in Deutschland bisher jedoch schleppend. Aktuell liegt Deutschland in der Homeoffice-Nutzung unterhalb des EU-Durchschnitts. Als Gründe werden oft Vorbehalte der Unternehmen und hohe Umstellungskosten genannt.

gebracht. Erste Umfrageergebnisse für Deutschland deuten auch darauf hin, dass viele Führungskräfte aufgrund der konkreten Lernerfahrungen in der Krise ihre Vorbehalte gegenüber Homeoffice abgebaut haben.[3]

Über die Chancen, die sich aus dieser Flexibilisierung ergeben, und die möglichen Risiken, die damit verbunden sind, wird aktuell viel diskutiert. So sehen viele Erwerbstätige die Chance auf eine bessere Vereinbarkeit von Beruf und außerberuflichen Tätigkeiten, wie beispielsweise die Sorgearbeit für Kinder und pflegebedürftige Familienangehörige, sie schätzen die Möglichkeit, den Arbeitsalltag nach den eigenen Bedürfnissen zu strukturieren, und die reduzierten Pendelzeiten zum Arbeitsplatz. Aus Sicht der Unternehmen lockt die Aussicht auf die Reduzierung der Kosten für Büroflächen. Gleichzeitig erschwert die räumliche Trennung der Beschäftigten aber den direkten Informationsaustausch, was sich negativ auf die Kreativität und arbeitsgruppenübergreifende Problemlösungen auswirken kann und angepasste Führungskonzepte erforderlich macht.

In diesem Beitrag nehmen wir eine Frage in den Blick, die in der Diskussion bisher wenig Beachtung gefunden hat: Welches Potenzial bieten Homeoffice-Arrangements, durch die räumliche Trennung der Arbeitsplätze vom Unternehmen die Beschäftigungsmöglichkeiten regional stärker zu streuen und damit Ungleichgewichte der regionalen Arbeitsmärkte auszugleichen? Wie unsere Analyse zeigt, ist das Potenzial beträchtlich, die konkrete Umsetzung wird aber aktuell durch die unzureichende Infrastruktur in der Fläche ausgebremst.

## Homeoffice-Potenzial: Welche und wie viele Arbeitsplätze sind homeoffice-fähig?

Um den Arbeitsausfall infolge staatlicher Kontakteinschränkungen im Zuge der COVID-19-Pandemie besser einschätzen zu können, haben in jüngster Zeit viele Studien das Homeoffice-Potenzial verschiedener Volkswirtschaften abgeschätzt.[4] Dabei wird zuerst das Homeoffice-Potenzial einzelner Berufe, etwa anhand von Tätigkeitsprofilen oder Umfragen bestimmt und dann auf Basis der Berufsstruktur der Volkswirtschaft das Homeoffice-Potenzial des Landes berechnet.

Die Studien unterscheiden sich primär in der Bestimmung des Homeoffice-Potenzials einzelner Berufe. Dingel und Neimann (2020) wählen einen konservativen Ansatz und bezeichnen diejenigen Berufe als homeofficefähig, die prinzipiell komplett im Homeoffice erledigt werden könnten. Dazu definieren sie Tätigkeitskomponenten als Ausschlusskriterien, die einer vollständigen Erledigung des Berufs im Homeoffice entgegenstehen, wie etwa Arbeit im Freien oder das Führen von Fahrzeugen. Auf Basis dieses konservativen Ansatzes wären in Deutschland etwas mehr als ein Drittel aller Arbeitsplätze vollständig homeofficefähig.

Alipour et al. (2020) bestimmen das berufsspezifische Homeoffice-Potenzial auf Basis der Erwerbstätigenbefragung des Bundesinstituts für Berufsbildung (BIBB) und der Bundesanstalt für Arbeitsschutz und Arbeitsmedizin (BAuA).[5] Die Erwerbstätigen werden unter anderem gefragt, ob sie das Angebot ihres Arbeitgebers, vorübergehend im Homeoffice zu arbeiten, annehmen würden. Diese Frage kann bejaht oder verneint, sowie mit dem Hinweis beantwortet werden, dass die Arbeit prinzipiell nicht im Homeoffice ausgeübt werden kann. Das Homeoffice-Potenzial eines Berufs wird als Anteil der Ja- und Nein-Antworten errechnet.

Alipour et al. (2020) bezeichnen also im Gegensatz zu Dingel und Neimann (2020) auch Berufe als homeofficefähig, die nur zum Teil im Homeoffice erledigt werden können. Das so ermittelte Homeoffice-Potenzial von etwa 56 % entspricht daher einer breiteren Definition der Homeoffice-Fähigkeit. Zusammen bilden beide Berechnungsmethoden eine Bandbreite, innerhalb derer das tatsächliche Homeoffice-Potenzial zu verorten ist.

## Homeoffice-Nutzung: Internationaler Vergleich

Gemäß dem European Labour Force Survey lag vor der Corona Krise im Jahr 2019 in Deutschland der Anteil der Beschäftigten, die „manchmal“ oder „normalerweise“ von zu Hause arbeiten, mit 13 % unterhalb des EU28-Durchschnitts (16 %) (vgl. Abbildung 1). Dabei gab es während der letzten zehn Jahre kaum Veränderungen in der Nutzungsrate. Demgegenüber verzeichnen Länder mit besonders intensiver Nutzung von Homeoffice im gleichen Zeitraum einen deutlichen Anstieg der Nutzungsraten. Insgesamt ist die Homeoffice-Nutzung in den einzelnen EU-Staaten sehr unterschiedlich stark ausgeprägt. In Ländern, in denen bereits vor der Corona-Krise eine intensive Nutzung von Homeoffice stattfand, hat ein besonders hoher Anteil an Beschäftigten während der Corona-Krise angefangen, im Homeoffice zu arbeiten (Eurofound, 2020). Sollte

3 So geben in einer aktuellen Befragung des Fraunhofer-Institut für Arbeitswirtschaft und Organisation IAO 42 % der Unternehmen an, das Angebot an entsprechenden Arbeitsmodellen auch nach der Krise ausweiten zu wollen, während 44 % noch unentschlossen sind und 13 % das Angebot nicht ausweiten wollen (Hoffmann et al., 2020). Evidenz zu positiven Erfahrungen mit Homeoffice in den USA liefert Ozimek (2020).

4 Für Deutschland wurde das Homeoffice-Potenzial etwa durch Alipour et al. (2020), Dingel und Neimann (2020) oder Fadinger und Schymik (2020) abgeschätzt. Für andere Länder schätzen Dingel und Neimann (2020) das Homeoffice-Potenzial zwischen 5 % für einige afrikanische Entwicklungsländer und rund 45 % für einige Länder in Europa. Luxemburg bildet mit einem Homeoffice-Potenzial von 55 % einen Ausreißer.

5 Diese Befragung wird vom Bundesinstitut für Berufsbildung (BIBB) und der Bundesanstalt für Arbeitsschutz und Arbeitsmedizin (BAuA) im sechsjährlichen Turnus durchgeführt und gibt Aufschluss über Anforderungen und Arbeitsumfeld von Berufen in Deutschland.

Abbildung 1

**Anteil der Beschäftigten im Homeoffice[1]**

[1] Anteil der Beschäftigten, die manchmal oder gewöhnlich im Homeoffice arbeiten. BG-Bulgarien, RO-Rumänien, CY-Zypern, LT-Litauen, HU-Ungarn, IT-Italien, LV-Lettland, GR-Griechenland, HR-Kroatien, ES-Spanien, SK-Slowakei, CZ-Tschechien, MT-Malta, DE-Deutschland, PL-Polen, PT-Portugal, EU-Europäische Union, SI-Slowenien, IE-Irland, EE-Estland, AT-Österreich, FR-Frankreich, BE-Belgien, UK-Großbritannien, DK-Dänemark, FI-Finnland, LU-Luxemburg, NL-Niederlande, SE-Schweden. [2] Keine Werte für 2009 verfügbar.

Quellen: Dingel und Neiman (2020) European labour force survey (EU-LFS); eigene Berechnungen.

sich dies nach der Krise verstetigen, wird sich die Nutzung von Homeoffice in Europa weiter auseinanderentwickeln.

Diese ausgeprägten internationalen Unterschiede in der Verbreitung von Homeoffice sind sowohl auf Unterschiede in der Branchen- und Tätigkeitsstruktur zwischen den Ländern, als auch in der technischen Ausstattung und Infrastruktur sowie auf kulturelle und institutionelle Unterschiede zurückzuführen. Zwar liegt der Beschäftigtenanteil im produzierenden Gewerbe in Deutschland deutlich über dem in den Niederlanden oder Schweden. Somit ist auch das tätigkeitsbedingte Potenzial des mobilen Arbeitens in Deutschland geringer (Berg et al., 2020; Özgüzel et al., 2020). Vergleicht man jedoch die tatsächliche Verbreitung von Homeoffice mit dem tätigkeitsbedingten Potenzial nach Dingel und Neimann (2020), wird deutlich, dass einige Länder mit einem ähnlich hohen Potenzial wie Deutschland eine höhere Verbreitung von Homeoffice aufweisen (vgl. Abbildung 1). Dies legt nahe, dass neben der Branchen- und Tätigkeitsstruktur auch andere Faktoren relevant für die Verbreitung von Homeoffice sind.

So liegt Deutschland im europäischen Vergleich seit Jahren bei der Verbreitung der für Homeoffice nötigen breitbandigen Internetanschlüsse sowohl bei Haushalten als auch bei Un-

Abbildung 2

**Verbreitung von Homeoffice und Breitbandversorgung[1] in der EU**

[1] Datenübertragungsrate von mindestens 100 Mbit/s. Verfügbare Anschlüsse bei Haushalten und Take-up-Rate bei Unternehmen. [2] Anteil der Beschäftigten, die 2019 manchmal oder gewöhnlich im Homeoffice arbeiteten.

Quellen: European labour force survey (EU-LFS); Eurostat.

Abbildung 3

**Homeoffice-Potenzial[1], Bevölkerungsdichte[2] und Baulandpreise[2] auf Kreisebene**

[1] Anteil der im jeweiligen Kreis sozialversicherungspflichtig Beschäftigten, die prinzipiell im Homeoffice arbeiten könnten. Stand: 2018. [2] Stand: 2017.

Quellen: Alipour et al. (2020); Bundesinstitut für Bau-, Stadt- und Raumforschung; Bundesamt für Karthographie und Geodäsie; eigene Berechnungen.

ternehmen zurück (vgl. Abbildung 2). Auch in der Ausstattung der Beschäftigten mit mobilen Endgeräten zeigen sich deutsche Unternehmen vergleichsweise zurückhaltend.

In deutschen Unternehmen besteht außerdem vielfach nach wie vor eine ausgeprägte Präsenzkultur. Umfragedaten für Deutschland aus dem Jahr 2015 lassen vermuten, dass eine Präferenz der Vorgesetzten für die Anwesenheit ihrer Beschäftigten das wichtigste Hemmnis des mobilen Arbeitens ist, noch vor der Eignung der Tätigkeiten oder der technischen Ausstattung (Arnold et al., 2015). Auch während der Corona-Krise erschwerten vor allem Vorbehalte der Führungskräfte und Geschäftsführungen sowie fehlende Betriebsvereinbarungen ein Ausweichen in die Arbeit von zu Hause (Hoffmann et al., 2020).

Schließlich belegt Deutschland bei der Digitalisierung der öffentlichen Verwaltung im Jahr 2020 nach wie vor einen Platz unter dem EU-Durchschnitt (Europäische Kommission, 2020). Zum einen hemmt dies die Verbreitung von Homeoffice im öffentlichen Dienst. Zum anderen mindern fehlende digitale Verwaltungsangebote auch für Unternehmen den Anreiz, Geschäftsprozesse zu digitalisieren und so bessere Voraussetzungen für die räumlich flexible Arbeit zu schaffen.

## Chancen verstärkter Homeoffice-Nutzung für regionale Arbeitsmärkte

Eine stärkere räumliche Trennung von Arbeitsort und Unternehmenssitz durch Arbeit im Homeoffice könnte die regionale Verteilung von Beschäftigungsmöglichkeiten erheblich flexibilisieren. Auf der Ebene der deutschen Landkreise sind die Möglichkeiten, Arbeitsplätze von Präsenzarbeit auf Homeoffice umzustellen, gemessen am Homeoffice-Potenzial nach Alipour et al. (2020) sehr heterogen verteilt (vgl. Abbildung 3, links). Das höchste Homeoffice-Potenzial hat die Stadt Köln mit knapp 68 % aller Arbeitsplätze, die zum Großteil ins Homeoffice verlegt werden könnten, das geringste Potenzial der Landkreis Sonneberg in Thüringen mit knapp 39 %. Zur Bestimmung des regionalen Homeoffice-Potenzials auf Landkreisebene wird dazu das Homeoffice-Potenzial einzelner Berufe herangezogen und mit der Zusammensetzung der Arbeitsplätze nach Berufen im jeweiligen Landkreis aggregiert.

Die Heterogenität des Homeoffice-Potenzials wird zu einem erheblichen Teil durch die unterschiedliche Wirtschaftsstruktur der Landkreise erklärt. Wirtschaftszweige im Dienstleistungsbereich weisen aufgrund ihrer Berufsstruktur ein deutlich höheres Homeoffice-Potenzial auf. Dementsprechend gibt es in Landkreisen mit einem hohen Dienstleistungsanteil, insbesondere solchen mit einem hohen Anteil wissensinten-

Abbildung 4

**Pendlersaldo[1], Homeoffice-Potenzial[2] und Breitbandversorgung[3] auf Kreisebene**

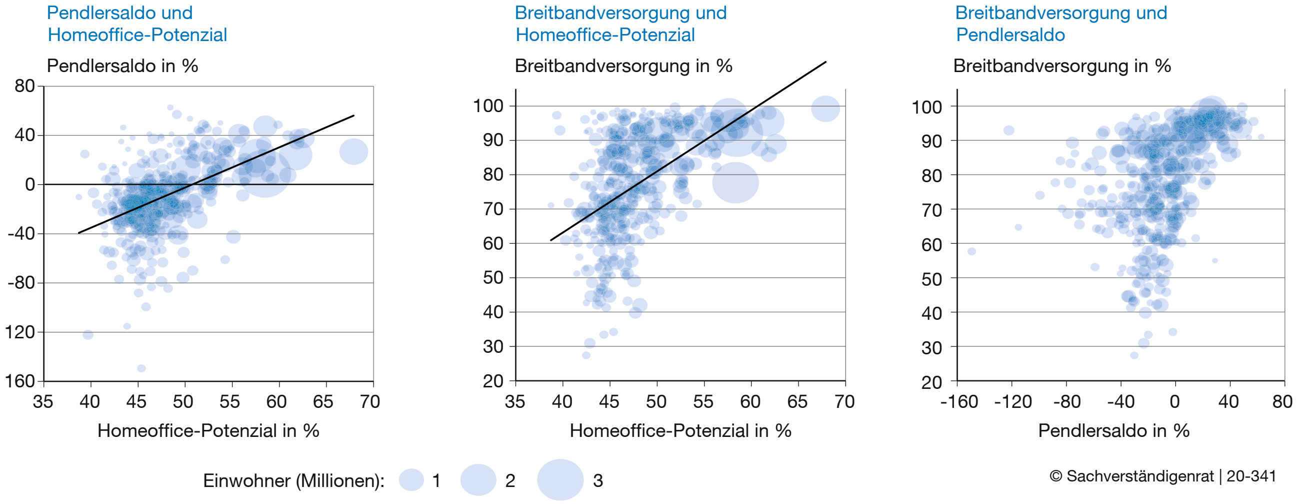

[1] (Einpendler – Auspendler)/sozialversicherungspflichtig Beschäftigte im jeweiligen Kreis. Stand: 2017. [2] Anteil der im jeweiligen Kreis sozialversicherungspflichtig Beschäftigten, die prinzipiell im Homeoffice arbeiten könnten. Stand: 2018. [3] Stand: 2017.

Quellen: Alipour et al. (2020); Bundesinstitut für Bau-, Stadt- und Raumforschung; eigene Berechnungen.

siver Dienstleistungen, die ein besonders hohes Homeoffice-Potenzial aufweisen, deutlich mehr Arbeitsplätze, die auf Homeoffice umgestellt werden könnten.

Unter den Kreisen mit hohem Homeoffice-Potenzial befinden sich viele Großstädte sowie Kreise in Ballungsgebieten mit einer hohen Bevölkerungsdichte (vgl. Abbildung 3, Mitte). In den vergangenen Jahrzehnten war eine zunehmende Divergenz der Entwicklung zwischen Ballungsräumen und dem ländlichen Raum zu beobachten, die sich beispielsweise in einer deutlich stärkeren Zunahme von Löhnen sowie Wohnkosten in Ballungsräumen niedergeschlagen hat. Diese Divergenz könnte durch eine verstärkte Nutzung von Homeoffice abgeschwächt werden. So ist das Homeoffice-Potenzial gerade in den Kreisen besonders hoch, die vergleichsweise hohe Preise für Bauland aufweisen, die sich in hohen Mietpreisen für Wohn- und Gewerbeimmobilien niederschlagen (vgl. Abbildung 3, rechts).

Neben der Entlastung von Ballungsgebieten in Hinblick auf knappes Bauland könnte eine verstärkte Nutzung von Homeoffice auch die Verkehrsinfrastruktur entlasten. Insbesondere in Kreisen, die einen positiven Pendlersaldo aufweisen, ist ein Großteil der Arbeitsplätze prinzipiell homeofficefähig (vgl. Abbildung 4, links). Diejenigen Beschäftigten, die derzeit längere Strecken zwischen Wohn- und Arbeitsort pendeln, dürften ein besonders großes Interesse daran haben, zukünftig einen Teil ihrer Arbeitszeit im Homeoffice zu verbringen.

Ob durch Homeoffice-Nutzung eine Verlagerung von Arbeitsplätzen aus den Ballungsgebieten heraus in die Fläche gelingen kann, hängt wesentlich von der Infrastruktur am Standort des Unternehmens und am Wohnort der Beschäftigten ab, insbesondere von der Verfügbarkeit von Breitbandinternet. Tatsächlich ist die Breitbandversorgung in Regionen mit Unternehmen, deren Tätigkeiten ein hohes Homeoffice-Potenzial aufweisen, tendenziell gut (vgl. Abbildung 4, Mitte). Einer Verlagerung der Homeoffice-Beschäftigung aus den Regionen mit positiven Pendlersalden heraus steht allerdings entgegen, dass die Regionen mit einem negativen Pendlersaldo eine schlechtere Versorgung mit Breitbandinternet haben (vgl. Abbildung 4, rechts). Gerade die Regionen, deren Bewohnerinnen und Bewohner durch die räumliche Trennung von Arbeitsplatz und Unternehmen profitieren könnten, dürften also aufgrund ihrer schlechteren Infrastrukturausstattung bei der Realisierung einer stärkeren Homeoffice-Nutzung gehemmt sein.

## Wirtschaftspolitische Implikationen

Um die Vorteile der Homeoffice-Nutzung realisieren und das Potenzial in der Breite der Regionen heben zu können, ist also eine Verbesserung des Breitbandzugangs, insbesondere auch außerhalb der Ballungsräume, eine zentrale Voraussetzung. Hier besteht im internationalen Vergleich ein erheblicher Nachholbedarf (Europäische Kommission, 2019). Eine Verbesserung könnte durch die Förderung von breitbandigem Internet in ländlichen Regionen erreicht werden.

Gelingt eine nachhaltige räumliche Flexibilisierung der Arbeit, eröffnen sich langfristig viele Chancen. Eine Abkehr von Präsenzpflichten kann die räumliche Entkopplung von Unternehmensstandort, Arbeitsplatz und Wohnort der Beschäftigten vorantreiben. Unternehmen könnten teure Büroflächen in Ballungszentren einsparen. Beschäftigte hätten die Möglichkeit, ihren Lebensmittelpunkt in den weiteren Umkreis der Stadt oder sogar außerhalb von Ballungszentren zu verlegen. Dort, wo dies schon der Fall ist, könnten Pendelzeiten reduziert werden. Insgesamt böten sich Entwicklungsmöglichkeiten in Regionen mit derzeit geringerer ökonomischer Aktivität und niedrigeren Lebenshaltungskosten, wodurch die regionale Ungleichheit reduziert würde. Eine solche Entwicklung müsste städteplanerisch begleitet werden. So würden sich nicht nur die Bedürfnisse an die Mobilitätsinfrastruktur ändern. Öffentliche Coworking-Spaces und Satellitenbüros könnten den Arbeitsplatz näher an den Wohnort der Beschäftigten bringen und einer sozialen Isolierung im Homeoffice entgegenwirken.

Gleichzeitig gilt es, möglichen Risiken einer Entgrenzung der Arbeit zu begegnen. Viele gesetzliche Regelungen zu Arbeitsschutz und Arbeitszeit sind in Hinblick auf einen Arbeitsplatz am Unternehmensstandort entstanden und dürften für die Arbeit im Homeoffice nicht mehr passgenau sein. So trägt der Arbeitgeber die Verantwortung für die technische Ausstattung und Ergonomie am Arbeitsplatz, kann diese im Homeoffice aber möglicherweise nicht überprüfen. Mindestruhezeiten stehen möglicherweise einer flexiblen, auf individuelle Bedürfnisse ausgerichteten Arbeitszeiteinteilung entgegen. Gleichzeitig darf Homeoffice nicht zu unbezahlter Mehrarbeit führen. Hier bedarf es einerseits klarer Regeln zum Schutz der Beschäftigten, andererseits muss geklärt werden, wie Arbeitgeber ihrer Pflicht zur Überprüfung der individuellen Arbeitszeit im Homeoffice nachkommen können.

Um das volle Potenzial räumlich flexibilisierter Arbeitsplätze zu nutzen, bedarf es also vielfältiger komplementärer Anstrengungen. Investitionen, die sich lohnen dürften.


Dr. Niklas Garnadt und Dr. Steffen Viete sind Mitarbeiter des Wissenschaftlichen Stabs des Sachverständigenrates.


Literatur

Alipour, J.-V., O. Falck und S. Schüller (2020), Germany’s capacities to work from home, CESifo Working Paper, 8227, CESifo Network.
Arnold, D., S. Steffes und S. Wolter (2015), Mobiles und entgrenztes Arbeiten, Forschungsbericht, Bundesministerium für Arbeit und Soziales.
Benkert D Arbeitsrechtliche Aspekte einer Tätigkeit im Home-Office NJW-Spezial 2019 2019 306 307
Berg, J., F. Bonnet und S. Soares (2020), Working from home: Estimating the worldwide potential, https://voxeu.org/article/working-home-estimating-worldwide-potential (1. September 2020).
Dingel, J. I. und B. Neiman (2020), How many jobs can be done at home?, Journal of Public Economics, im Erscheinen.10.1016/j.jpubeco.2020.104235 PMC7346841 32834177
Eurofound (2020), Living, working and COVID-19: first findings, April 2020.
Europäische Kommission (2020), Digital Economy and Society Index (DESI) 2020, Digital public services, https://ec.europa.eu/digital-single-market/en/desi (1. September 2020).
Europäische Kommission (2019), Broadband Coverage in Europe 2018 Mapping progress towards the coverage objectives of the Digital Agenda, https://ec.europa.eu/newsroom/dae/document.cfm?doc_id=62760 (1. September 2020).
Fadinger, H. und J. Schymik (2020), The effects of working from home on covid-19 infections and production — a macroeconomic analysis for germany, CRC TR 224 Discussion Paper, 167, Project B 06, Universität Bonn und Universität Mannheim.
Hoffmann, J., A. Piele und C. Piele (2020), Arbeiten in der Corona-Pandemie — auf dem Weg zum New Normal, Studie des Fraunhofer IAO in Kooperation mit der Deutschen Gesellschaft für Personalführung DGFP, http://publica.fraunhofer.de/eprints/urn_nbn_de_0011-n-5934454.pdf (1. September 2020).
Möhring, K. et al. (2020), Die Mannheimer Corona-Studie: Schwerpunktbericht zu Erwerbstätigkeit und Kinderbetreuung, German Internet Panel (GIP), Universität Mannheim.
Nilles J Telecommunications and organizational decentralization IEEE Transactions on Communications 1975 23 10 1142 1147 10.1109/TCOM.1975.1092687
Özgüzel, C., P. Veneri und R. Ahrend (2020), Potential for remote working across different places, OECD COVID-19 Policy Note.
Ozimek, A. (2020), The Future of Remote Work, https://ssrn.com/abstract=3638597 (1. September 2020).
Schröder, C. et al. (2020), Vor dem Covid-19-Virus sind nicht alle Erwerbstätigen gleich, DIW aktuell, 41.
